# YouTube as a Reliable Source of Patient Education for Peyronie's Disease: A Quality Assessment Study

**DOI:** 10.7759/cureus.114066

**Published:** 2026-08-06

**Authors:** Mohammed Saad, Abbas Abuelhassan, Mujahid Abuagla Dafalla Hamed, Abdulrahman Mohammed

**Affiliations:** 1 Urology, Barts Health NHS Trust, London, GBR; 2 Obstetrics and Gynaecology, Ashford and St. Peter's Hospitals NHS Foundation Trust, Surrey, GBR; 3 Faculty of Medicine, Al Neelain University, Khartoum, SDN; 4 Orthopaedics, Sultan Bin Abdul Aziz Humanitarian City, Riyadh, SAU

**Keywords:** erectile dysfunction, peyronie’s, youtube study, youtube© video analysis, youtube videos

## Abstract

Introduction: Peyronie's disease (PD) is a penile connective tissue disorder characterized by fibrous plaque formation within the tunica albuginea, resulting in penile curvature, pain, deformity, and impaired sexual function. Due to embarrassment and limited awareness, many patients seek health information online, with YouTube becoming a widely used source of medical education. However, the quality and reliability of PD-related YouTube content remain unclear. This study aimed to evaluate the quality, reliability, and educational value of YouTube videos related to PD and assess their suitability as a patient information resource.

Methods: A YouTube search was conducted on May 20, 2026, using five predefined search terms related to PD. The first 20 videos from each search were screened. Videos were included if they were English-language, patient-oriented educational videos lasting between 1 and 20 minutes. Video characteristics were recorded, and content quality was assessed using the DISCERN instrument, Global Quality Scale (GQS), and Journal of the American Medical Association (JAMA) Benchmark Criteria. Statistical analysis included one-sample t-tests and Spearman's rank correlation analysis.

Results: Of 100 screened videos, 12 met the inclusion criteria. The mean video duration was 4:03 ± 3:08 minutes, with a mean of 212,275 ± 440,000 views. The mean total DISCERN score was 51.42 ± 9.59, indicating lower-range good-quality information, with no significant difference from the score of 51, which is the good-quality threshold (p = 0.883). Individual DISCERN domains, compared to the midpoint score of 3, demonstrated statistically significantly higher scores for clarity of aims (3.92 ± 1.08, p = 0.0137), achievement of aims (3.67 ± 0.98, p = 0.0388), and relevance to patients (3.83 ± 0.94, p = 0.0105). The mean GQS score of 2.92 ± 1.38 was consistent with moderate educational quality and did not differ significantly from the reference score of 3 for moderate-quality educational content (p = 0.838). The mean JAMA Benchmark Criteria score was 1.50 ± 1.23, indicating low reliability. No significant correlations were identified between DISCERN, GQS, and JAMA scores.

Conclusion: YouTube videos regarding PD provide generally acceptable but inconsistent educational value and reliability. While many videos effectively address patient concerns, important deficiencies remain in treatment-related information, transparency, and evidence-based content. Patients should be encouraged to critically evaluate online information, and clinicians should guide individuals toward reliable educational resources. Further development of transparent, comprehensive, and patient-centered digital resources is required to improve online education for men with PD.

## Introduction

Peyronie's disease (PD) is a disorder of the penis characterized by the formation of localized fibrous plaques within the tunica albuginea, which may be palpable beneath the penile skin [1,2]. Although PD is thought to develop secondary to repetitive trauma, many patients do not recall a specific traumatic event preceding symptom onset [3]. The condition predominantly affects men over the age of 45 years, with additional risk factors including a positive family history and associated connective tissue disorders [3,4]. Clinical manifestations vary depending on the disease phase, with symptoms differing between the acute and chronic stages [5]. Common presentations include penile pain, progressive penile curvature, and penile deformity, which may negatively impact sexual function and contribute to erectile dysfunction [6]. Management of PD depends on disease severity, duration, and patient-specific factors, with treatment options including conservative medical therapies and surgical intervention for selected cases [2,7].

Despite its significant impact on quality of life, many patients with PD experience discomfort or embarrassment when seeking medical advice or discussing their symptoms with healthcare providers, family members, or sexual partners [8]. Limited awareness of available diagnostic and therapeutic options may further contribute to delayed presentation and management [9,10]. Consequently, patients frequently seek information from online sources as either a primary or supplementary resource before or after consultation with a healthcare professional [11,12]. Among digital platforms, YouTube has become one of the most widely used sources of medical information worldwide due to its accessibility, free availability, and ability to communicate complex medical concepts through visual and narrative formats [13,14]. For sensitive or stigmatized conditions such as PD, video-based platforms may be particularly appealing, allowing patients to access health information privately and at their own convenience [15].

However, YouTube lacks a formal peer-review system, and uploaded videos originate from a heterogeneous range of content creators, including healthcare professionals, patients, and commercial entities, resulting in variable quality, accuracy, and reliability of the information presented [16,17]. Several studies have evaluated YouTube as a source of patient education across various urologic conditions, with varying findings regarding the quality, reliability, accuracy, and transparency of available content [18,19]. Despite the growing patient reliance on YouTube for health-related information, the quality and educational value of videos specifically addressing PD have not been evaluated.

Given the sensitive nature of PD and its potential impact on psychological well-being and sexual function, ensuring access to accurate, comprehensive, and transparent educational resources is essential to support informed patient decision-making [10]. Therefore, this study aimed to evaluate the quality, reliability, and educational value of YouTube videos related to PD and assess their suitability as an information resource for patients.

## Materials and methods

A YouTube search was conducted on May 20, 2026, to identify videos providing patient-oriented educational information on PD. Searches were performed without signing into a personal account, and browser history was cleared prior to the search in order to reduce the influence of personalized recommendations and algorithm-based video selection.

The search was conducted using the following predefined keywords: "Peyronie's disease", "Peyronie disease", "What is Peyronie's disease", "Peyronie's disease treatment", and "Peyronie's disease symptoms". For each search term, the first 20 videos retrieved were screened, resulting in an initial pool of 100 videos.

Videos were included if they were publicly available, in English, had a duration of at least one minute, and contained educational content intended for patients with PD. Eligible videos included information related to the definition of PD, symptoms, diagnosis, treatment options, or prognosis.

Videos were excluded if they were primarily intended for healthcare professionals, were duplicates, or did not contain relevant educational information regarding PD. Videos longer than 20 minutes were also excluded because the study aimed to evaluate commonly accessed patient-oriented educational videos rather than comprehensive professional lectures.

**Figure 1:**
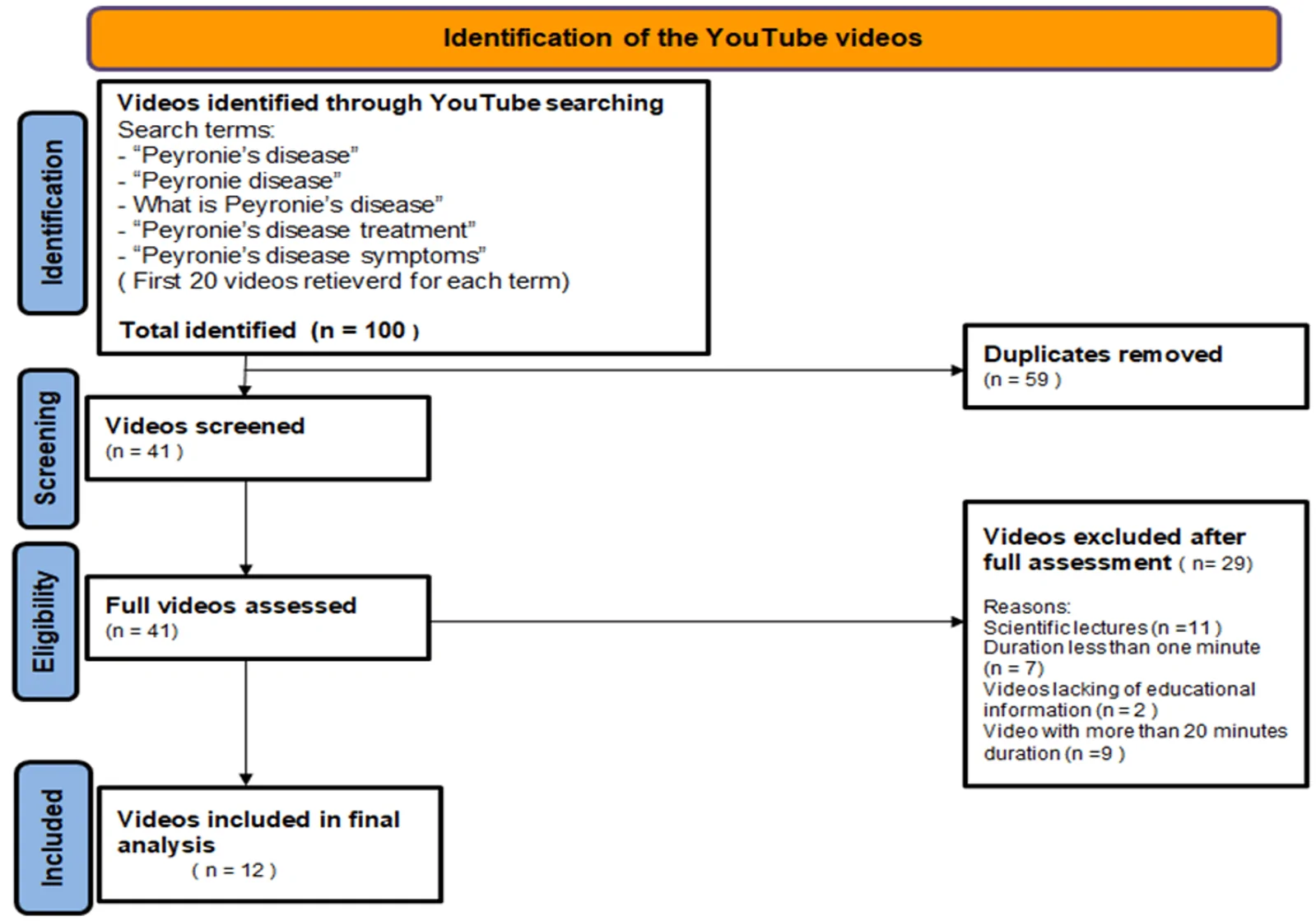
Flow diagram illustrating the identification, screening, and inclusion of YouTube videos related to Peyronie’s disease in the study.

Although 100 videos were initially screened, only 12 fulfilled the strict inclusion criteria because many videos were duplicates, aimed at healthcare professionals, exceeded the duration limit, or lacked patient-focused educational content (Figure 1). For each included video, the upload date, duration, number of views, and number of likes were recorded. 

The DISCERN instrument was used to evaluate the reliability and quality of treatment-related information provided in the included videos and is defined as a validated 16-item questionnaire comprising three sections: reliability of the publication (questions 1-8), quality of information regarding treatment choices (questions 9-15), and an overall quality rating (question 16) [20]. Each item is scored on a five-point Likert scale, with total scores ranging from 16 to 80, and higher scores indicate better quality and reliability of information. The total DISCERN scores were categorized as very poor (16-26), poor (27-38), moderate (39-50), good (51-62), and excellent (63-80) [20].

The overall educational value and usefulness of each video were assessed using the Global Quality Scale (GQS), a validated five-point scale used to evaluate online medical information, with scores assigned as follows: 1, poor quality with insufficient information and limited educational value; 2, generally poor quality with some relevant information but major deficiencies; 3, moderate quality with acceptable educational content but lacking important details; 4, good quality with useful and comprehensive information suitable for patients; and 5, excellent quality with highly informative, well-structured, and valuable educational content [21].

The reliability and transparency of the included videos were assessed using the Journal of the American Medical Association (JAMA) Benchmark Criteria, which is a validated tool that evaluates four domains of online medical information quality: authorship, attribution, disclosure, and currency [22]. Each criterion is scored as present (1 point) or absent (0 points), with total scores of 3 to 4 points classified as high reliability and scores of 0 to 2 points classified as low reliability [22].

All included videos were independently reviewed and scored by two reviewers. Discrepancies in scoring or interpretation between reviewers were resolved through discussion and consensus. The final scores used for analysis represented the agreed assessment between the two reviewers.

Continuous variables were expressed as mean (M) ± standard deviation (SD). Mean and SD were calculated for each DISCERN item, and a one-sample t-test was performed for each item against the midpoint value of 3. A one-sample t-test was also used to compare the overall mean DISCERN score with the reference value of 51, which represents the established threshold for good-quality and reliable health information. Similarly, the mean GQS score was compared with the reference value of 3, which represents moderate educational quality. Results for the JAMA Benchmark Criteria were presented as M ± SD.

Spearman's rank correlation analysis was performed to assess relationships among the quality assessment instruments. Correlations were evaluated between DISCERN and GQS scores to determine the association between information reliability and overall educational quality; between DISCERN and JAMA Benchmark Criteria scores to examine the relationship between information quality and transparency indicators; and between JAMA Benchmark Criteria scores and GQS ratings to assess whether transparency indicators were associated with overall educational quality.

A p-value <0.05 was considered statistically significant. All statistical analyses were performed using GraphPad Prism version 10 (GraphPad Software, LLC, San Diego, California).

## Results

The included videos demonstrated a mean time since upload of 3.72 ± 2.32 years and a mean duration of 4:03 ± 3:08 minutes. The videos received a mean of 212,275 ± 440,000 views, with view counts ranging from 3,900 to 1.6 million. Similarly, the mean number of likes was 789 ± 1,523, ranging from 0 to 5,600 likes.

**Figure 2:**
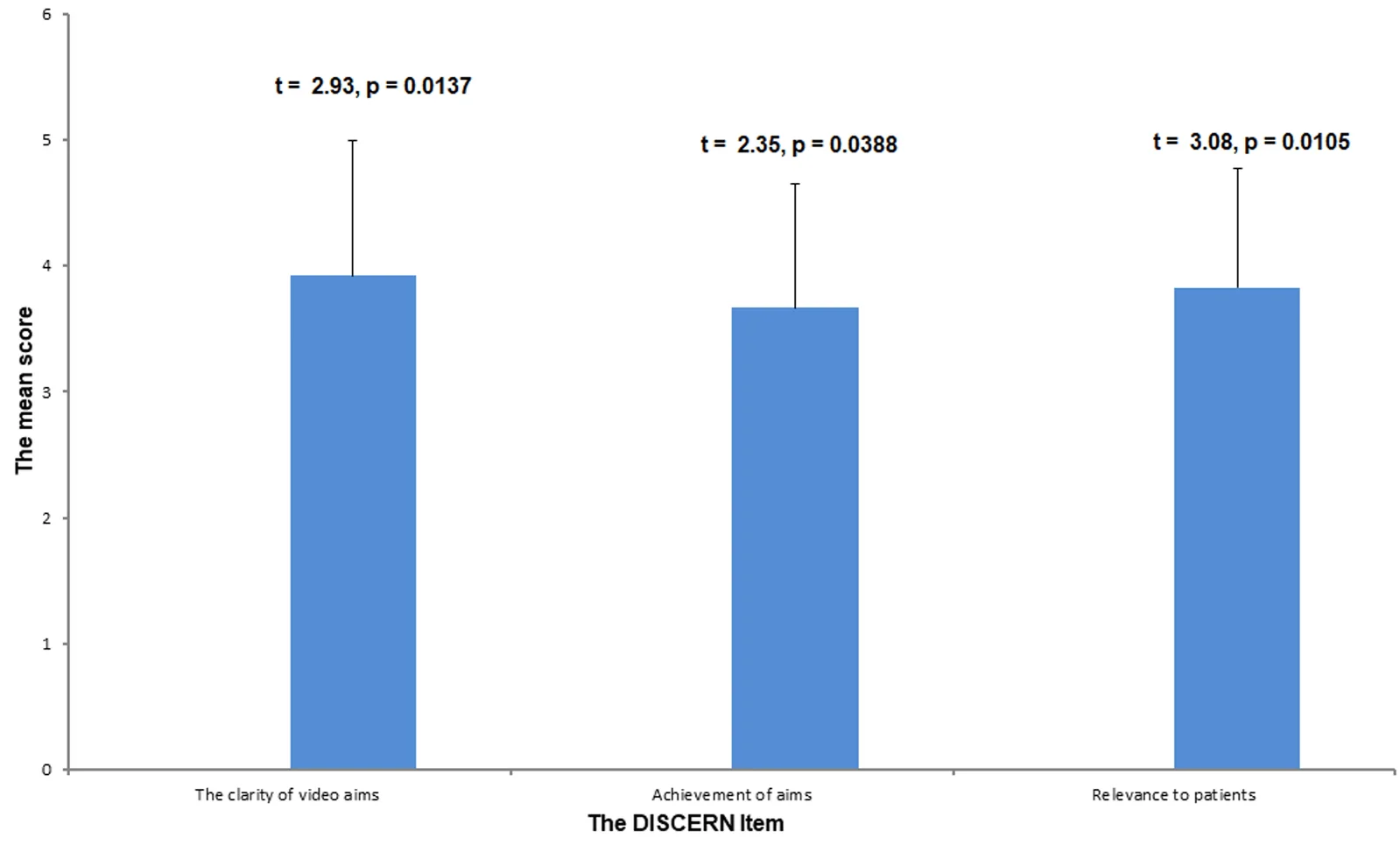
DISCERN items showing statistically significant higher scores. One-sample t-test results for DISCERN items 1, 2, and 3, with the observed scores compared against the predefined midpoint score of 3.

The mean scores for individual DISCERN items ranged from 2.67 ± 1.15 to 3.92 ± 1.08. Compared with the predefined midpoint score of 3, statistically significantly higher scores were observed for clarity of the video's aims (3.92 ± 1.08, p = 0.0137), achievement of those aims (3.67 ± 0.98, p = 0.0388), and relevance to patients (3.83 ± 0.94, p = 0.0105) (Figure 2). The remaining DISCERN domains did not demonstrate statistically significant differences from the midpoint value of 3 (Table 1). 

**Table 1:** DISCERN domain scores showing no significant difference from the midpoint value of 3. A one-sample t-test was used to compare each DISCERN item against the midpoint score 3.

DISCERN item	Assessment domain	Mean ± SD	t	p-value
D4	The sources of information are described	2.83 ± 1.11	-0.52	0.615
D5	The information is balanced and unbiased	2.92 ± 1.24	-0.23	0.820
D6	Additional sources are listed	3.33 ± 1.15	1.00	0.339
D7	The video provides current and up-to-date information	3.00 ± 1.65	0.00	1.000
D8	The video explains how each treatment functions	2.67 ± 1.15	1.00	0.339
D9	The video outlines the benefits of each treatment option	3.00 ± 1.35	0.00	1.000
D10	The video outlines the potential risks and side effects of each treatment option	3.25 ± 1.14	0.76	0.465
D11	The video explains the potential consequences of not receiving treatment	2.83 ± 1.47	-0.39	0.704
D12	The video describes how choices affect quality of life	3.42 ± 1.08	1.35	0.204
D13	The video makes clear that multiple treatment options are available	3.08 ± 1.62	0.18	0.862
D14	The video supports shared decision-making	3.08 ± 1.08	0.26	0.798
D15	The video mentions areas of uncertainty	3.25 ± 1.18	0.74	0.477
D16	The video overall quality rating	3.33 ± 1.37	0.84	0.420

In terms of information transparency, the evaluated parameters demonstrated scores comparable to the moderate reference level, including the description of information sources (2.83 ± 1.11, p = 0.615) and impartiality of information (2.92 ± 1.24, p = 0.820). No statistically significant differences were identified for the listing of additional sources (3.33 ± 1.15, p = 0.339) or the delivery of timely and updated information (3.00 ± 1.65, p = 1.000).

Regarding treatment-related information, scores for outlining the function of each treatment (2.67 ± 1.15, p = 0.339), describing the benefits of each treatment option (3.00 ± 1.35, p = 1.000), the potential risks and side effects of each treatment option (3.25 ± 1.14, p = 0.465), and the possible effects of remaining untreated (2.83 ± 1.47, p = 0.704) were not significantly different from the midpoint value.

Domains assessing the influence of decision-making on quality of life (3.42 ± 1.08, p = 0.204), the existence of more than one treatment option (3.08 ± 1.62, p = 0.862), encouraging patient involvement in treatment decisions (3.08 ± 1.08, p = 0.798), highlighting areas of uncertainty (3.25 ± 1.18, p = 0.477), and the overall quality (3.33 ± 1.37, p = 0.420) also showed no statistically significant differences from the reference value.

The mean total DISCERN score was 51.42 ± 9.59, and the mean GQS score was 2.92 ± 1.38. Neither DISCERN scores nor GQS ratings differed significantly from their respective reference values (t(11) = 0.15, p = 0.883, and t(11) = -0.21, p = 0.838, respectively). Spearman's rank correlation analysis demonstrated no significant correlation between DISCERN and GQS scores (ρ = 0.014, p = 0.965). The mean total JAMA Benchmark Criteria score was 1.50 ± 1.23 (Table 2). 

**Table 2:** Characteristics of the videos included in the study.

Video No.	Time since upload	Duration	Views	Number of likes	Total DISCERN score	Total JAMA score	GQS
1 [23]	9 years	3:43	519,000	1,700	36	2	3
2 [24]	4 years	4:55	66,000	397	34	1	2
3 [25]	4 years	1:58	20,000	122	40	3	2
4 [26]	1 year	13:30	39,000	663	60	3	5
5 [27]	10 months	3:19	3.900	24	55	0	4
6 [28]	6 years	1:30	150,000	0	48	0	3
7 [29]	5 years	3:45	21,000	99	58	1	4
8 [30]	4 years	5:05	1,600,000	5,600	55	3	5
9 [31]	2 years	4:05	31,000	146	56	1	3
10 [32]	9 months	1:23	4.400	12	62	0	1
11 [33]	3 years	1:19	19000	73	57	1	1
12 [34]	5 years	4:00	74000	631	56	3	2

Correlation analysis revealed no significant association between DISCERN and JAMA Benchmark Criteria scores (ρ = -0.315, p = 0.318). Similarly, no significant correlation was observed between JAMA Benchmark Criteria scores and GQS ratings (ρ = 0.298, p = 0.347) (Table 3). 

**Table 3:** Correlation analysis between the assessment tools. GQS: Global Quality Scale.

Comparison	Spearman’s ρ	p-value
Overall DISCERN and GQS	0.014	0.965
Total DISCERN scores and JAMA Benchmark Criteria	−0.315	0.318
JAMA Benchmark Criteria scores and GQS	0.298	0.347

## Discussion

The overall mean DISCERN score in this study indicated that YouTube videos related to PD were within the lower range of good-quality information and did not significantly differ from the predefined reference threshold. These findings suggest that available videos generally provide an acceptable level of reliability and educational value; however, they do not consistently exceed established standards for high-quality patient information [27,30,31]. Although YouTube may serve as a useful initial resource for individuals seeking information about PD, patients should remain aware of variability in content quality and avoid relying on the platform as their sole source of medical guidance [13].

Analysis of individual DISCERN items demonstrated that videos performed significantly better than the midpoint value in terms of clarity of aims, achievement of those aims, and relevance to patient concerns. This suggests that most PD-related videos were appropriately focused on patient interests and successfully communicated their intended purpose [26,31]. This is particularly relevant given that accessibility, convenience, and the ability to present information in an engaging format are key factors contributing to patients' preference for video-based health resources over traditional written materials [18,35]. However, despite adequate communication of general information, treatment-related domains, including explanations of treatment functions, potential benefits, risks, side effects, and the consequences of remaining untreated, did not demonstrate significant improvement beyond the midpoint value [23,25]. These findings indicate that while YouTube PD-related videos may effectively introduce patients to the condition, they frequently lack the comprehensive treatment-related information required to support informed decision-making. This limitation is particularly important in PD, where management decisions depend on disease severity, patient expectations, treatment risks, and individual preferences [5,16,36].

The educational value assessed using the GQS was comparable to a moderate-quality standard, indicating that the comprehensiveness and educational usefulness of available YouTube PD-related videos remain inconsistent. Furthermore, the absence of a significant correlation between DISCERN and GQS scores suggests that perceived educational value and information reliability do not necessarily coincide [32]. A video may be visually engaging, accessible, and perceived as useful while lacking sufficient accuracy, depth, or balanced information [19]. Conversely, a reliable source may not always present information in a format that optimally supports patient understanding [16]. Therefore, video popularity, accessibility, or perceived usefulness should not be considered substitutes for objective measures of information quality [37].

Assessment of transparency and reliability using the JAMA Benchmark Criteria revealed further limitations in the available PD-related YouTube content. The mean JAMA score corresponded to the low-reliability category, reflecting deficiencies in key transparency domains. Although YouTube provides an accessible platform for PD, the absence of consistent transparency regarding content creators, information sources, and evidence supporting medical claims may compromise the credibility of available material [13,18]. Furthermore, the lack of significant correlations between JAMA scores and either DISCERN or GQS ratings suggests that transparency indicators are not necessarily associated with overall reliability or educational quality [29,33]. This highlights the importance of evaluating online health resources using multiple quality dimensions rather than relying on a single assessment measure [16,35].

From a patient-centered perspective, these findings highlight the need for YouTube videos on PD that provide evidence-based information, transparent sourcing, and effective communication. Given the sensitive nature of PD and potential barriers to seeking medical advice, reliable online video educational resources may support patient awareness and decision-making. High-quality videos developed by recognized professionals may help address current gaps, as similarly reported in a previous study evaluating YouTube videos for erectile dysfunction [15].

The characteristics of the included videos further illustrate the variability of YouTube as a patient education platform for PD. Although some videos had been available for several years and achieved substantial viewer engagement, older content may not reflect evolving evidence, updated treatment recommendations, or changes in clinical practice [12,23]. Additionally, the relatively short duration of many videos may limit their ability to address the complex nature of PD, including diagnosis, treatment options, outcomes, and psychosocial considerations [19,33].

This study has several limitations. First, the relatively small number of eligible videos may limit the generalizability of the findings; however, this also reflects the limited availability of patient-oriented PD educational content on YouTube. Second, although assessments were performed independently by two reviewers, subjective variation in interpretation and scoring cannot be completely excluded. Third, only English-language videos were evaluated, and therefore the findings may not represent the quality of PD-related information available in other languages or regions. Finally, YouTube is a dynamic platform where content is continuously uploaded, modified, or removed, meaning that the availability and quality of videos may change over time.

## Conclusions

This study demonstrates that YouTube videos on PD provide an acceptable level of reliability and educational value; however, the overall quality of available content remains variable and does not consistently exceed established standards. Although many videos successfully address patient-relevant topics and present clear objectives, important gaps remain in the provision of comprehensive treatment-related information, discussion of risks and alternatives, and overall educational depth. Limitations in transparency, including inadequate disclosure of authorship, sources, and information currency, further affect the credibility of available content. Given the widespread use of YouTube as a source of health information, patients with PD should be encouraged to critically evaluate online resources and avoid relying solely on video-based information when making decisions regarding management. Clinicians can play an important role in guiding patients toward accurate, evidence-based, and comprehensive educational resources. Future initiatives focused on developing high-quality, transparent, and patient-centered videos may improve the value of YouTube as an educational platform for individuals with PD. Future studies should include larger sample sizes, involve multiple independent reviewers to improve assessment reliability, include patient comprehension assessments, evaluate content across multiple languages, and explore additional online platforms to provide a more comprehensive understanding of the quality and accessibility of digital health information on PD.
